# What is a disease? What is the disease clinical benign prostatic hyperplasia (BPH)?

**DOI:** 10.1007/s00345-019-02691-0

**Published:** 2019-02-25

**Authors:** Keong Tatt Foo

**Affiliations:** Department of Urology, Singapore General Hospital, Academia Level 5, 20 College Road, Singapore, 169856 Singapore

**Keywords:** Benign prostatic hyperplasia, Definition, Grading, Staging, Transabdominal ultrasound, Individualised care

## Abstract

**Introduction:**

Though clinical benign prostatic hyperplasia (BPH) is a common disease worldwide, there is still much confusion in the literature and the many clinical guidelines as to its definition. Often the disease is associated with lower urinary tract symptoms (LUTS) and managed according to only symptoms. This leads to undertreatment in some patients with severe bladder outlet obstruction (BOO) with no symptoms, and overtreatment in patients with LUTS but no clinical BPH.

**Definition of a Disease:**

Fundamentally, a disease can be defined as an abnormal structure or function or a condition which may cause harm to the organism.

**Definition of clinical BPH:**

Thus, clinical BPH can be defined as prostate adenoma/adenomata, causing a varying degree of BOO, which may eventually cause harm to the patients. With this definition, we are then able to differentiate the disease clinical BPH from the many other less common causes of LUTS, and then treat it according to its severity.

**Diagnosing clinical BPH:**

Clinical BPH can be diagnosed with non-invasive ultrasound in the clinic, grading it according to the shape (intravesical prostatic protrusion) and size of the prostate.

**Clinical Significance:**

Treatment can then be planned according to the disease severity using our staging system that classifies severity according to the presence or absence of significant obstruction and bothersomeness of symptoms.

**Conclusion:**

This would lead to better individualised and cost-effective management of the disease clinical BPH.

## Introduction

The term benign prostatic hyperplasia (BPH) has been reserved for histological diagnosis, and the term benign prostatic enlargement (BPE) is used to describe the disease as seen in the clinic [1]. However, small prostates can also cause obstruction and symptoms [2]. And more recently, this common disease has been termed as benign prostatic obstruction (BPO) where the cause of bladder outlet obstruction (BOO) is thought to be due to the prostate [3]. But is BPO a disease?

## Definition of a disease

A disease can be defined as an abnormal homeostasis in structure or function or a condition that may result in harm to the organism. High blood sugar per se is not diabetes mellitus. It is the result and not the cause of the disease. Rather, the cause is abnormal insulin metabolism of the islet cells. Similarly, BPO is the consequence and not the cause of clinical BPH; the cause is prostate adenoma/adenomata [4].

## Defining clinical BPH

Thus, clinical BPH can be defined as prostate adenoma/adenomata causing BOO that may cause harm to the bladder and eventually the kidneys. Our study has shown that obstruction depends more on the site than size of the adenoma [5]. A small adenoma at the bladder neck forming the middle lobe can cause significant obstruction to the bladder due to distortion of the funnelling effect of the normal bladder neck. On the other hand, an adenoma forming the lateral lobes can grow to a considerable size before causing obstruction by compressing the prostatic urethra. In flow dynamics, compression is less obstructive than distortion [4, 6].

Due to the slow progression of the disease, patients may get used to the symptoms and not have any complaints. They may not have lower urinary tract symptoms (LUTS) and present instead with sudden acute retention of urine or less commonly be found to have chronic retention with renal failure. Some may present with painless gross haematuria, or urinary infection. With the introduction of PSA in health screening, many patients may present with raised PSA.

Therefore, clinical BPH can be defined as prostate adenoma/adenomata causing a varying degree of BOO, with or without symptoms.

## Diagnosing clinical BPH

With the above definition, clinical BPH can nowadays be diagnosed with non-invasive ultrasound by measuring intravesical prostatic protrusion (IPP) in the clinic. IPP is measured in the sagittal view of a comfortably full bladder (about 200 ml) and it is the distance in mm from the innermost protrusion of the prostate perpendicularly down to the base at the circumference of the bladder [7]. It has a 100% positive predictive value and 100% specificity for diagnosing prostate adenoma [5]. Together with uroflowmetry, prostate adenoma can be ruled out as a cause of LUTS if there is no IPP and the flow rate is good [8]. This is useful in the differential diagnosis of male LUTS.

Prostate-specific antigen (PSA) is also important in the differential diagnosis. In patients with no clinical BPH, no prostatitis and no prostate cancer, PSA is generally less than 1 μg/l [6]. This is seen in patients after transurethral enucleation of the prostate adenoma/adenomata) [9]. PSA is related to the size of the adenoma and in early studies investigating normal PSA levels, many of the subjects might have prostate adenoma of varying sizes with no LUTS symptoms, leading to PSA less than 4 μg/l being accepted as normal and the misconception that PSA increases with age. In contrast, we have observed in our clinic that if a patient has no clinical BPH (adenoma), no prostatitis and no cancer, the PSA level remains the same over the years [6].

## Clinical relevance

With the new definition of clinical BPH, further assessment is done to determine whether there is possible harm to the bladder and kidneys. When the voiding function is affected, persistent post-void residual urine of more than 100 ml is observed, whereas when the storage function is affected, the maximum voided volume is low, less than 100 ml [10]. Both parameters can be easily measured in the clinic. Obstruction is considered as significant when the above dysfunctions are present. The severity of the symptoms can be assessed with the International Prostate Symptoms Score (IPSS) and the quality of life (QoL) index. The QoL index is more important than IPSS and a QoL score of 3 and above is considered as bothersome [11].

Thus the severity of clinical BPH can be staged accordingly:

Stage I: no significant obstruction and no bothersome symptoms;

Stage II: no significant obstruction but has bothersome symptoms;

Stage III: significant obstruction irrespective of symptoms; and

Stage IV: complications of clinical BPH such as retention of urine, recurrent haematuria, urinary tract infection, and bladder stones.

Prostate adenoma/adenomata can be graded according to IPP (grade 1: ≤ 5 mm, grade 2: > 5 mm–10 mm, and grade 3: > 10 mm) and prostate volume (*a*: ≤ 20 g, *b*: > 20–40 g, and *c*: > 40 g). Grade 1a prostates are least obstructive and grade 3a prostates most obstructive, and the disease can be managed accordingly [12]. Grade 3a prostates would generally be better treated surgically, as they are small and easily removed by transurethral resection whereas grade 1c prostates would be more suitable for medical treatment with five-alpha reductase inhibitors.

About 60% of the patients with clinical BPH have low-grade and low-stage disease and they can be watched with advice on fluid adjustment, diet, and exercise while those with higher stage and higher grade disease would need more aggressive medical or surgical treatment options [13, 14].

For patients with discordant grade and stage, urodynamic studies may still be necessary to assess obstruction and underactive detrusor dysfunctions, especially if more invasive treatment is contemplated. This discordance occurs in the minority, as shown in a study of 408 patients [13]. Of 44 patients in stage III, 37 patients had grade 3 IPP; only seven patients (16%) had an IPP grade less than 3.

Overactive bladder (OAB) may be suspected from storage symptoms of urgency and frequency. If a patient has a high grade IPP of 3 (more than 10 mm) and low maximum voided volume (less than 100 ml), he should be offered treatment with options for surgery or five-alpha reductase inhibitors if the prostate is more than 30 g. Anticholinergics may cause more harm to patients, increasing post-void residual urine or even leading to retention of urine [10].

In another study of 114 patients with pressure flow study done, IPP was the best predictor for BOO, with the positive predictive value at 72% compared to PV and PSA at 65% and 68%, respectively [15].

## Conclusion

The main objective of this review is to elucidate the definition of the disease clinical BPH. The focus is not on LUTS/BPH, which has been reviewed previously by our group [16, 17] and the other authors [18].

In medical school, we are taught to treat the disease, and not symptoms. Yet, currently many international guidelines still manage this common condition of male LUTS/BPH according to symptoms, using the International Prostate symptoms score (IPSS). Likewise, pharmaceutical and medical device companies use IPSS to gauge patients’ response to treatment.

With the definition that clinical BPH is essentially a benign neoplasm (adenoma/adenomata), which may progress and eventually cause harm to the patient due to obstruction, BPO would be more important than the symptoms (IPSS). Therefore, obstruction should be given more weight in decision making in treating the disease according to severity. This can be done by the staging classification based on the presence or absence of significant obstruction and bothersomeness of symptoms. With a proper definition, the disease can be diagnosed and classified accordingly and treated appropriately.

Apart from history and physical examination, diagnosis in the clinic can be done more precisely with non-invasive ultrasound and the disease classified according to the grade of IPP to predict its course, as in malignant neoplasm. This would help to individualise management of the disease in the clinic, while also taking into account the patient’s age, comorbidity and preferences for cost-effective personalised care.
